# Successful Cardiac Surgical Management in a Trisomy 21 Child After Long-Term Hospitalization Associated With Bronchopneumonia and Hepatitis C Virus Seropositivity: A Case Report

**DOI:** 10.7759/cureus.61309

**Published:** 2024-05-29

**Authors:** Vishal V Bhende, Rahul Tandon, Mathangi Krishnakumar, Rupal M Patel, Ashwin S Sharma

**Affiliations:** 1 Pediatric Cardiac Surgery, Bhanubhai and Madhuben Patel Cardiac Centre, Shree Krishna Hospital, Bhaikaka University, Karamsad, IND; 2 Pediatrics, Pramukhswami Medical College, Shree Krishna Hospital, Bhaikaka University, Karamsad, IND; 3 Anaesthesiology, St John's Medical College Hospital, Bengaluru, IND; 4 Microbiology, Pramukhswami Medical College, Shree Krishna Hospital, Bhaikaka University, Karamsad, IND; 5 Internal Medicine, Gujarat Cancer Society Medical College, Hospital and Research Centre, Ahmedabad, IND

**Keywords:** ventricular septal defect, trisomy 21, selective pulmonary vasodilators, respiratory infections, pulmonary arterial hypertension, pneumonia, hepatitis c virus (hcv), eisenmenger syndrome, down syndrome, bronchopneumonia

## Abstract

A 31-month-old girl with trisomy 21 (Down syndrome) was seen in the emergency department of pediatrics because of oxygen desaturation associated with features of lower respiratory tract infections. She was born at full term and diagnosed with congenital heart disease (CHD) having ventricular septal defect (VSD), and patent ductus arteriosus (PDA); consequently, she underwent corrective surgery after adequate optimization of treatment. Incidentally, she was detected to have the presence of anti-hepatitis C virus (HCV) antibodies. In this case report, we mainly focus on the multi-modal approach to medical and surgical management.

## Introduction

Trisomy 21 (T21), also referred to as Down syndrome (DS), is acknowledged as the most prevalent chromosomal anomaly, manifesting in approximately 1 in 800-1000 live births [1,2]. This condition is linked with congenital heart disease (CHD) in 40-45% of instances, predominantly atrioventricular septal defects (40-45%), followed by ventricular (30%) and atrial septal defects (15%) [3]. Remarkably, the past half-century has seen a significant decline in infant mortality among those with T21, largely due to advancements in cardiac surgical interventions for early correction of heart abnormalities [4].

Despite these advancements, some children with T21 who do not undergo early cardiac defect repair reach adulthood and develop severe pulmonary arterial hypertension (PAH) as a result of persistent shunt defects, known as Eisenmenger syndrome (ES) [5]. At this advanced stage, intracardiac repair becomes unviable, often leading families to believe that no further treatment options exist. However, the use of selective pulmonary vasodilators has shown promise in enhancing exercise capacity and potentially improving outcomes in patients with ES, including those with T21 [6,7].

DS, characterized by an additional 21st chromosome, is a genetic anomaly detectable from conception, resulting in 47 chromosomes instead of the typical 46. It affects roughly 1 in every 800 newborns worldwide, with an estimated total affected population of 40 to 50 million [8]. Children with this syndrome often exhibit hypotonia, increased joint flexibility, predisposition to obesity, short limbs, and delays in neurological and language development. About 40-50% of these children are diagnosed with CHD, underscoring the need for personalized physiotherapy and psychotherapy [8].

Before the establishment of routine blood screening for HCV, children who underwent surgery for CHD were at a considerable risk of contracting the infection [9]. For individuals with DS, chronic hepatitis C often stems from transfusions received during previous heart surgeries [9,10]. Moreover, DS is frequently associated with a variety of immunological disorders, including abnormal peripheral blood lymphocyte composition, cellular dysfunction, and autoimmunity [11].

## Case presentation

The study protocol and ethical issues were reviewed and approved by The Institutional Ethics Committee (IEC-2) of H.M. Patel Centre for Medical Care and Education, Anand, Gujarat on April 5, 2024 (Approval No. IEC/BU/2024/Cr. 17/118/2024). The authors confirm that this case report does not contain any patient data that can be used to identify individuals. Given that the subject was a minor with T21, written consent was duly acquired from the parents.

A 31-month full-term girl was brought to the trauma and emergency care (TEC) department of Shree Krishna Hospital (SKH), Karamsad with complaints of fever, cough, and cold for two days associated with vomiting and difficulty in breathing for 1 day. Due to episodes of desaturation, the child was kept on nasal prongs oxygen support with 4 liters/min and then it was hiked up to 10 liters/min on a non-rebreather mask (NRBM).

The child weighed 9.5 kg on admission, was afebrile and a blood pressure recording of 89/53 mm Hg was recorded with a respiratory rate (RR)of 46/min. The child had a previous history of difficulty in breathing when the baby was 2 months old. DS was diagnosed and confirmed by karyotyping and a 2D echocardiogram revealed patent ductus arteriosus (PDA) and a wide ventricular septal defect (VSD). At that time, she was found to be in heart failure, and anti-failure medications were prescribed.

In the present admission, the child continued to have features of bronchopneumonia and was being treated accordingly. Her laboratory values were monitored from the day of admission (Day 1) till she was optimized for cardiac surgical intervention (Day 63; Table 1).

**Table 1:** Summary of the patient’s laboratory data on admission till surgical intervention

Sr. No.	Test	Patient Results								Reference Range
		Day 01	Day 03	Day 09	Day 22	Day 26	Day 32	Day 56	Day 61	
01	Hemoglobin	9.1	8.5	8.9	12.0	10.7	11.2	9.9	9.9	11.1-14.1 g/dl
02	Total leucocyte count (WBC Count)	24.9	13.8	18	17	9	18	11.4	10.2	6-16 X 1000 /ul
03	C-reactive protein (CRP)	68.1	55.4	21.5	17.2	12.4	5.6	4.3	22.4	<3 mg/l
04	Red blood cell count (RBC)	4.23	4.03	3.84	4.93	4.55	4.80	4.17	4.10	million/cu.mm.
05	Platelet count	130	191	-	252	341	214	289	309	200-550 x 1000/ul
06	Differential count									
	Neutrophils	90	62	74	72	34	76	37	52	25-50%
	Lymphocytes	08	33	21	21	57	20	54	42	50-85%
	Eosinophils	01	01	01	02	03	01	02	02	2-4%
	Monocytes	00	00	00	00	00	00	00	00	5-9%
	Basophils	00	00	00	00	00	00	00	00	0-1%
07	Serum thyroid-stimulating hormone (TSH)	1.24	-	-	-	-	-	-	-	1.30-1.90 mIU/l
08	Serum creatinine	0.33	0.27	-	-	0.38	0.35	-	0.28	0.20-0.40 mg/dl
09	Serum electrolytes									
	Serum sodium	136	136	133	-	-	133	134	139	136-145 mmol/l
	Serum potassium	4.1	4.1	3.8	-	-	4.7	4.5	4.1	3.8-5.2 mmol/l
	Serum chloride	102	103	96	-	-	98	95	98	95-105 mmol/l
10	HCV antibody and HCV RNA by polymerase chain reaction (PCR)							Anti-HCV antibodies: Positive (enzyme-linked fluorescent assay)	HCV RNA PCR (qualitative) – Target not detected	

**Figure 1:**
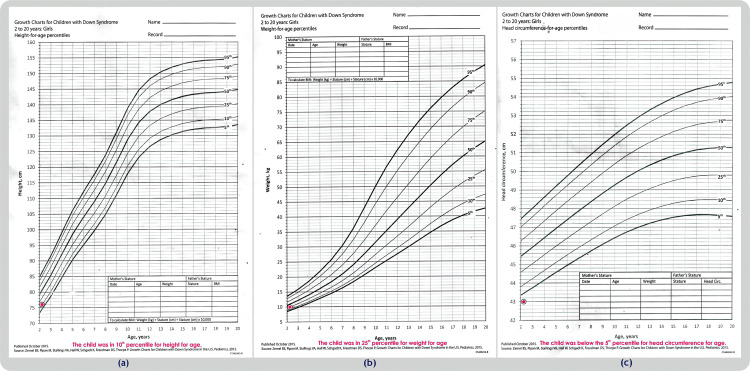
Charts depicting growth patterns for children diagnosed with Down syndrome. (a) Height-for-age percentiles, (b) weight-for-age percentiles, (c) head circumference-for-age percentiles. Image Credits : Dr. Vishal V. Bhende

The child exhibited developmental delays and a growth trajectory that consistently fell below the expected benchmarks from birth (Figure 1).

**Figure 2:**
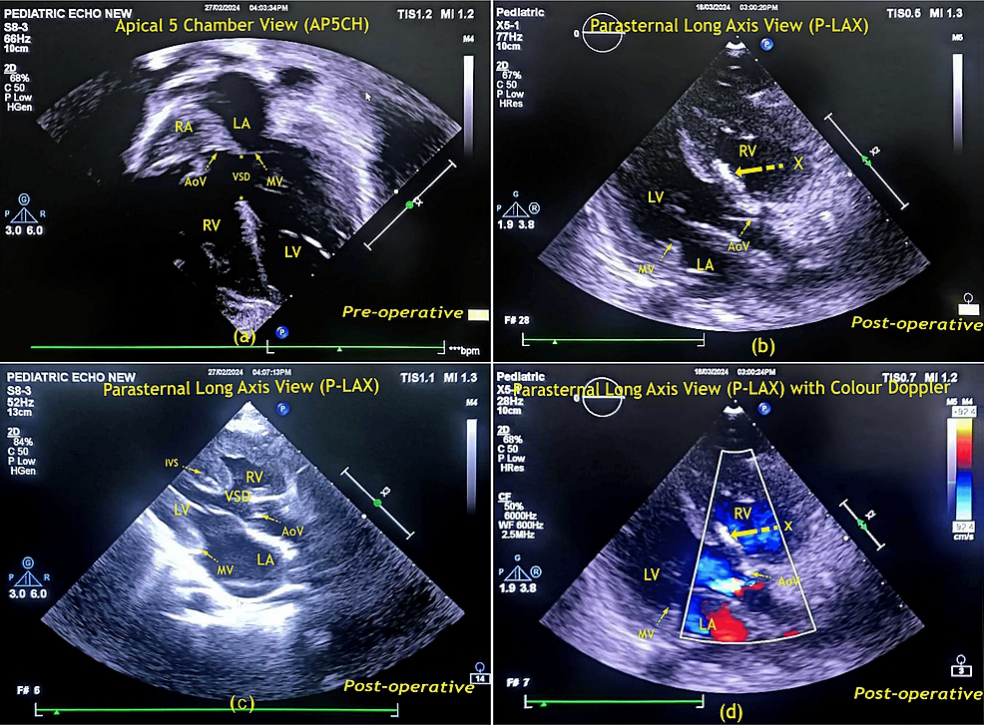
2D trans-thoracic echocardiography findings. (a) pre-operative view, (b-d) postoperative view RA: right atrium; LA: left atrium; AoV: aortic valve; MV: mitral valve; RV: right ventricle; LV: left ventricle; IVS: inter-ventricular septum; VSD: ventricular septal defect; X: VSD closed with glutaraldehyde-treated autologous pericardial patch. Image credits: Dr. Mahesh H. Bhatt/Mr. Naresh J. Fumakiya

As the child had features of bronchopneumonia and rising total counts, appropriate antibiotics were considered, though still, the child continued to be dependent on oxygen support of 2 liters/min nasal prongs. Her anemia was corrected with 1 unit of blood transfusion and qualitative molecular analyses were negative for SARS-CoV2 (COVID-19). Her 2-D echocardiography revealed a large inlet VSD, moderate-sized PDA, predominantly left-to-right shunt, and evidence of severe hyperkinetic PAH (Figure 2).

**Figure 3:**
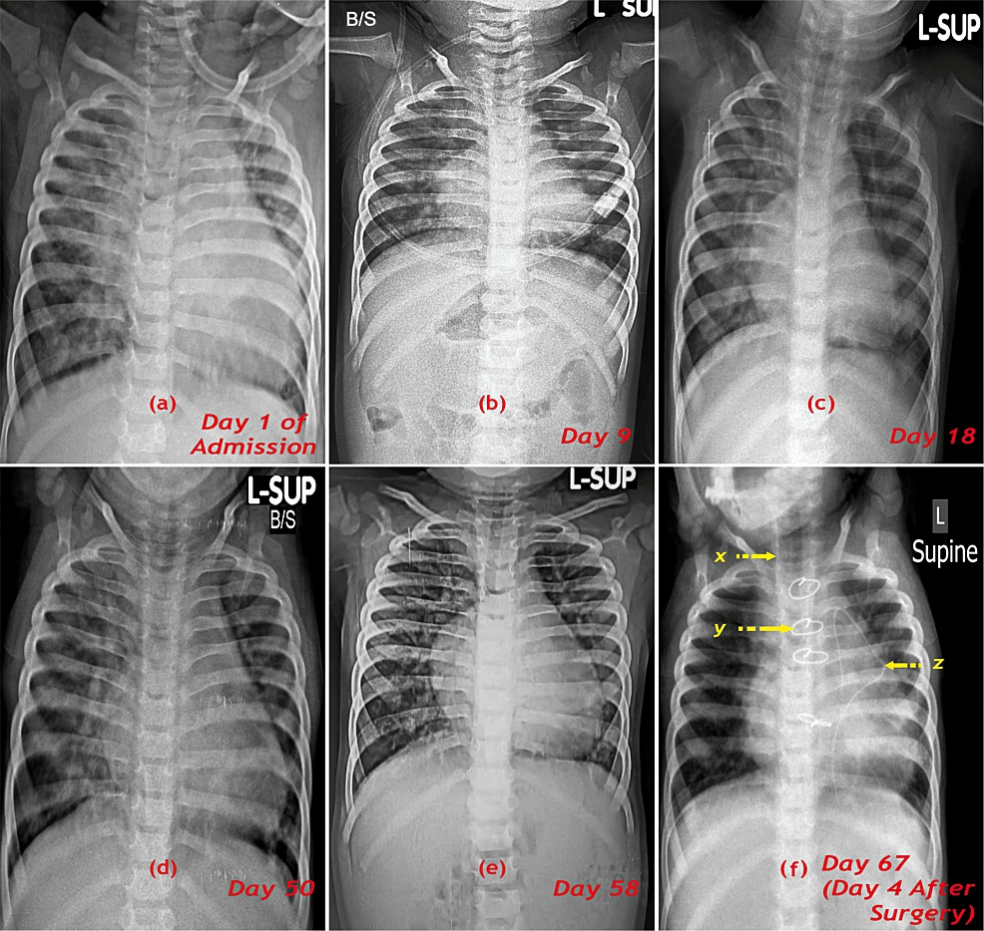
Chest X-rays at various stages from the day of admission till post-cardiac surgery depicting the resolution of bronchopneumonia. x: central venous pressure line; y: sternal wires; z: pacing wire Image credits: Dr. Vishal V. Bhende

**Figure 4:**
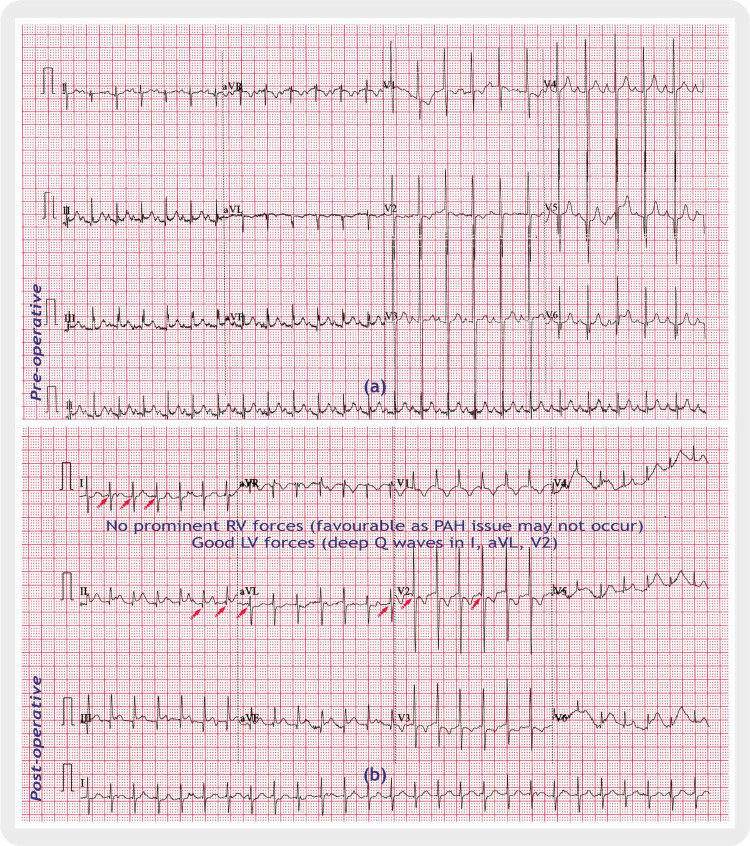
Electrocardiograms (ECGs). (a) pre-operative, (b) postoperative Deep Q waves are marked with red arrows in I, aVL, V2 in (b) postoperative panel. Image credits: Dr. Mahesh H. Bhatt

Following the resolution of serial chest X-rays from the day of admission till the date of surgery and baseline electrocardiograms (ECG), which didn’t reveal any arrhythmia (Figures 3-4).

**Figure 5:**
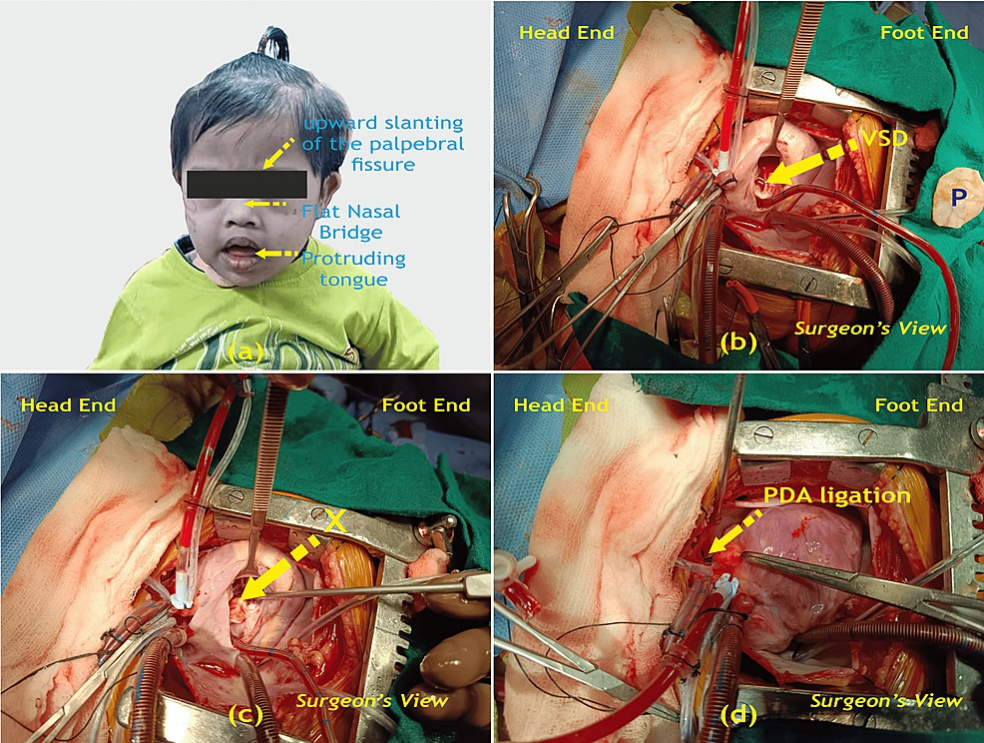
(a) Trisomy 21 (T21) child with features, (b), (c), and (d) intraoperative view of pre-closure and post-closure of VSD with PDA ligation. VSD; ventricular septal defect; P: glutaraldehyde-treated pericardial patch; PDA: patent ductus arteriosus. X: glutaraldehyde-treated pericardial patch closure of VSD. Image credits: Dr. Vishal V. Bhende

The child was planned for VSD closure with an autologous glutaraldehyde-treated pericardial patch and ligation of PDA with universal precautions adopted in view of HCV infection (Figure 5).

The cardiopulmonary bypass (CPB) time was 103 minutes, and the aortic cross-clamp (ACC) time was 52 minutes. Arterial blood pressure remained relatively stable pre- and post-bypass, while right ventricular and main pulmonary artery pressures significantly decreased post-bypass. The 2D echocardiogram showed a small residual VSD and PDA with left-to-right shunts, along with mild aortic regurgitation and moderate tricuspid regurgitation. Mild PAH was noted, and bi-ventricular function remained good, with no pericardial effusion. The surgery was uneventful with no post- or intraoperative complications (Table 2).

**Table 2:** Intra- and postoperative parameters ACC: aortic cross-clamp; AR: aortic regurgitation; CPB: cardiopulmonary bypass; FiO_2_: fraction of inspired oxygen; LA left atrium; LV: left ventricle; MPA: main pulmonary artery; RA: right atrium; RV: right ventricle, SO_2_: saturations of oxygen; PA: pulmonary artery; PAH: pulmonary arterial hypertension; PDA: patent ductus arteriosus; P/M: peak/mean; VSD: ventricular septal defect

Sr. No.	Parameters	Values
01	CPB time	103 min
02	ACC time	52 min
03	RA and PA SO_2_ RA & FiO_2_ 50%	RA 47%; PA 49%
04	Arterial blood pressure (ABP)	Pre-bypass-87/36(59) mmHg. Post-bypass-88/35(55) mmHg
05	RV Body	Pre-bypass-72/06 (40) mmHg. Post-bypass-35/10 (20) mmHg
06	MPA	Pre-bypass-72/40 (57) mmHg. Post-bypass-35/17 (20) mmHg
07	2D echocardiography	A visible VSD patch observed in situ, dilated LA and LV, small residual VSD with left-to-right shunt, VSD gradient ~ 62 mmHg, small residual PDA with left-to-right shunt, PDA gradient (P/M) 72/34 mmHg, mild AR, AR jet deceleration time ~ 480 msec, vena contracta width of AR Jet ~3 mm, moderate tricuspid regurgitation, mild PAH, no pericardial effusion, good bi-ventricular function.

Results

The patient had a significant recovery after VSD repair with PDA ligation. There were no complications related to surgery and the child was discharged having a cardiac surgical intensive care unit (CSICU) stay of 6 days and a hospital stay of 74 days. The child recovered in sinus rhythm and was advised for health supervision parameters viz. (a) monitoring growth patterns, especially body mass index (BMI), (b) obtain annual ear-specific audiologic evaluation; brainstem evoked response audiometry (BERA), (c) evaluation of the eye every 2 years, (d) measure thyroid-stimulating hormone (TSH) annually.

## Discussion

In the contemporary medical landscape, the proactive treatment and surgical correction of CHD in individuals with DS have become standard procedures. However, this approach represents a significant evolution from past practices. The clinical characteristics of DS were initially identified by John Langdon Down in 1866, and it wasn’t until 1959 that Jerome Lejeune established the connection between DS and the chromosomal anomaly known as T21 [12].

**Figure 6:**
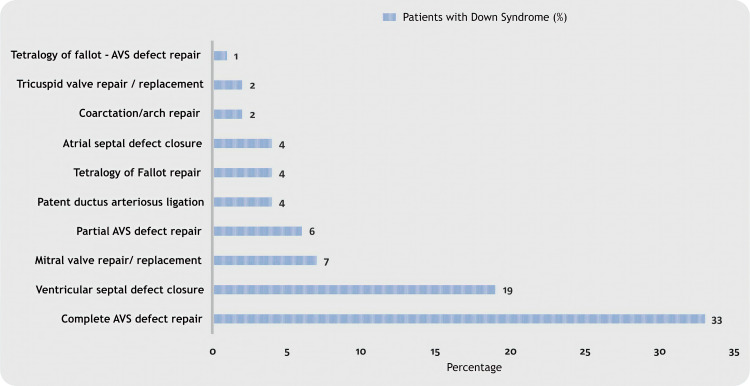
The most frequently performed cardiac surgeries in individuals with Down syndrome, as outlined in the STS-CHS Database. STS-CHS: Society of Thoracic Surgeons Congenital Heart Surgery; AVS: atrioventricular septal

Open heart procedures for addressing congenital heart anomalies became predominantly accessible during the 1960s and the initial years of the 1970s (Figure 6).

In the nascent stages of heart surgery for congenital defects, there was a prevalent trend of not considering patients with DS for cardiac surgical interventions [13]. As medical management for non-cardiac ailments in DS infants saw improvements and the success rates for complete cardiac repairs of major septal defects in infancy rose, it became apparent that DS patients could successfully undergo surgical procedures. This shift in medical understanding led to a change in societal views, and consequently, several DS infants with CHD started receiving surgical referrals. Currently, it is a widely accepted practice to propose cardiac surgery for DS patients with CHD who meet the general criteria for such interventions.

Furthermore, children with DS exhibit a heightened susceptibility to respiratory infections, both upper and lower, and the frequency of recurrent respiratory tract infections can profoundly impact their well-being, resulting in a higher likelihood of hospital admissions, the need for more intensive care, and an elevated mortality risk [14]. The subject of our study presented with symptoms of bronchopneumonia, which was managed successfully with meticulous respiratory care and the administration of the appropriate antibiotics. This approach to managing bronchopneumonia, alongside the surgical rectification of cardiac anomalies, facilitated a more rapid recovery in the post-surgical phase and a diminished length of hospital stay. The subject was also found to be positive for anti-HCV antibodies. The Centers for Disease Control and Prevention have advocated for universal precautions in handling blood and bodily fluids since 1987, with ongoing updates to these guidelines. In cases involving known HCV-infected patients undergoing surgery, it is preferable to schedule their surgery as the last procedure of the day, limiting the presence to essential personnel only. Minimizing the risk of injuries from sharps can be achieved through establishing a neutral zone for passing instruments, employing needle-free devices, staples, and blunt sutures for closing deep tissues. Comprehensive barrier precautions, including the use of plastic aprons, face protection, and water-resistant gowns, as well as double-gloving, can significantly reduce blood exposure by 87% and the volume of transmitted blood by 95%, thus lowering the potential viral load from an infected patient [15].

Later evaluations revealed the child’s HCV RNA Qualitative PCR test to be negative, indicating no detectable target. This outcome implies that patients testing positive for HCV antibodies but negative for HCV RNA, in the absence of recent or ongoing HCV transmission risks, are generally not considered to have an active HCV infection, and usually, no further action is deemed necessary [16]. Testing is required with a different HCV antibody assay to differentiate between past, resolved HCV infection from biologic false-positivity for HCV antibody. However, in specific scenarios, such as suspected recent HCV infection, clinical symptoms of HCV, or concerns regarding the integrity of the specimen, additional testing may be advised, including a follow-up HCV RNA test and appropriate counseling [17].

Another aspect that needs to be considered during peri-operative management of these patients is the presence of hypothyroidism. It is the most common endocrine disorder affecting up to 8% of children with DS [18]. In our patient, we evaluated for this in the pre-operative period, and was part of our follow-up protocol. Though our patient was euthyroid, recognizing and treating correctable causes of growth retardation is imperative to ensure a favorable outcome following cardiac surgery.

## Conclusions

The case study presented illustrates an instance of congenital anomalies linked to DS, particularly a VSD and a PDA. Furthermore, HCV infection should be confirmed by repeat HCV antibody tests and/or HCV RNA along with regular monitoring of liver function tests and other treatment objectives. The long-term outcomes for these children are predominantly influenced by the timely surgical repair of cardiac anomalies, rather than the direct management of DS.
